# Supplementary material for: Unraveling the regulative development and molecular mechanisms of identical sea urchin twins
Source: Nat Commun. 2025 Sep 5;16:8005. doi: 10.1038/s41467-025-63111-z (PMC12413439; doi:10.1038/s41467-025-63111-z)
Supplement: Supplementary file 1 — Supplementary Information [file 41467_2025_63111_MOESM1_ESM.pdf]

Supplementary Information

Unraveling the regulative development and molecular mechanisms of identical sea urchin twins

Haruka Suzuki, Junko Yaguchi, Koki Tsuyuzaki, *Shunsuke Yaguchi

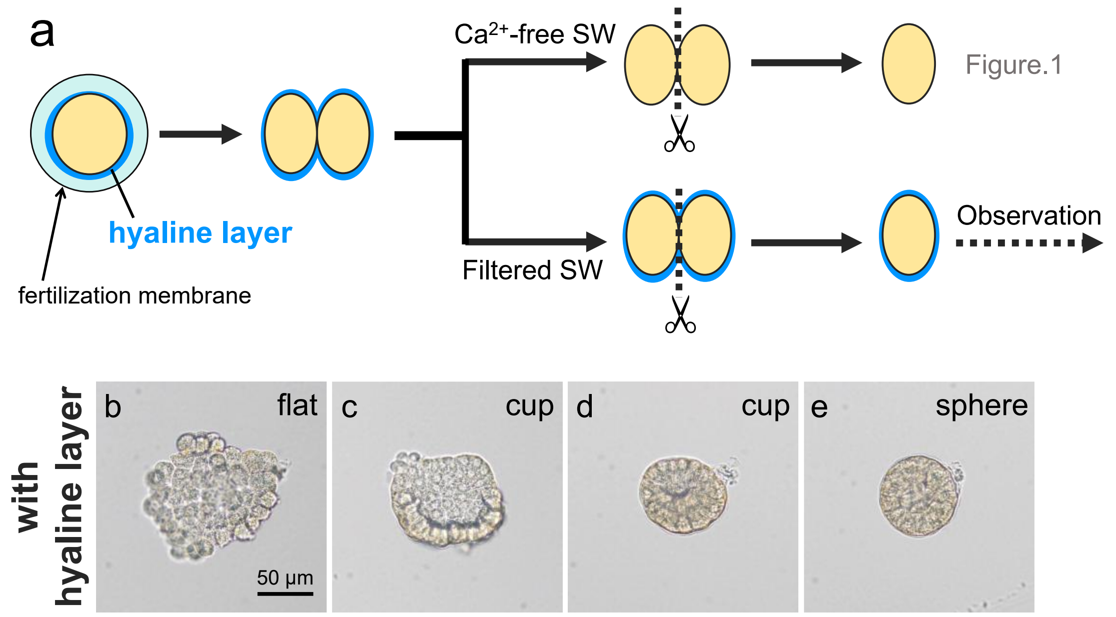

Figure S1 | Hyaline layer does not affect the developmental pattern of halved embryos. (a) Schematic image of experimental procedure. At 2-cell stage, fertilization envelope was removed and the blastomere was isolated in filtered seawater (SW). Developmental process of hyaline-less halved embryos with Ca^{2+}-free SW treatment is shown in Figure 1. (b-e) Developmental process of the halved embryos with hyaline layer. No developmental process difference was observed between halved embryos with and without hyaline. Developmental stage of each image is written in right corner.

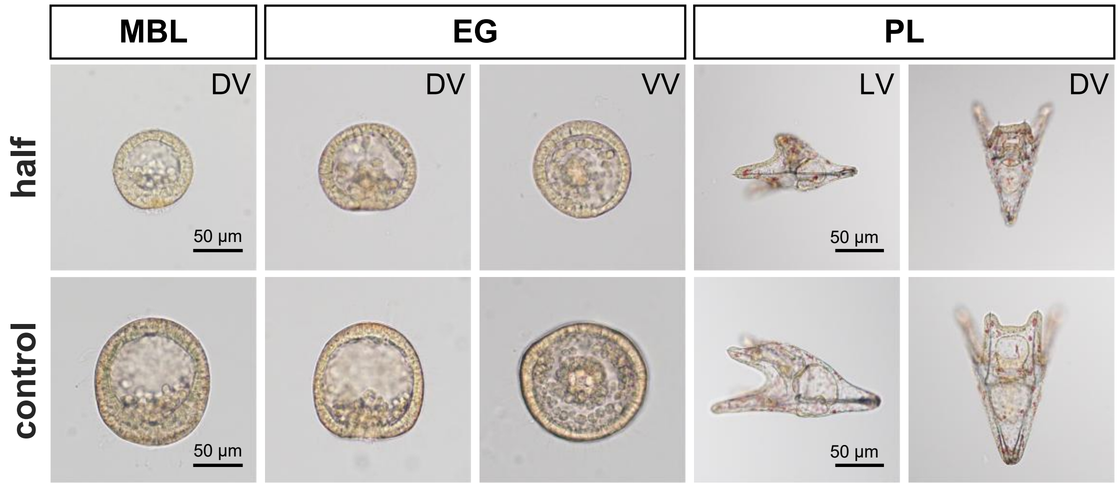

Figure S2 | Developmental process of halved embryos after blastula stage. Halved embryos developed as intact embryos after becoming blastula. MBL, mesenchyme blastula; EG, early gastrula; PL, pluteus larvae; DV, dorsal view; VV, vegetal view; LV, lateral view.

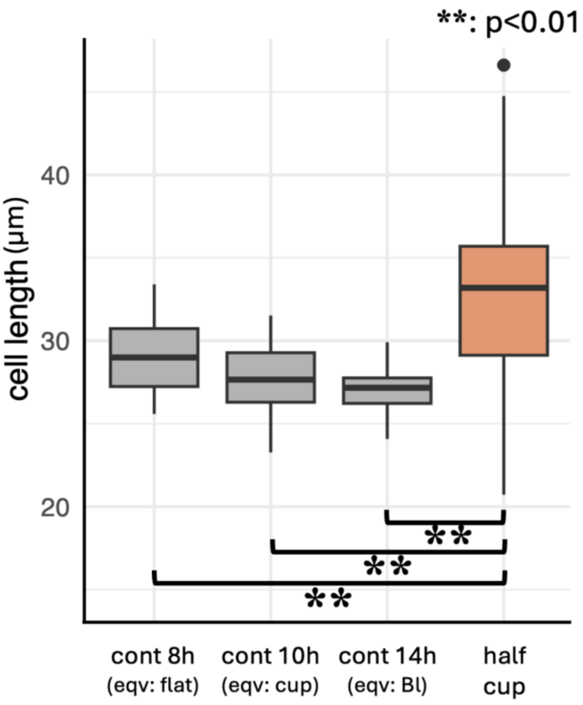

Figure S3 | Cell length comparison along apical-basal axis between control blastula and cup shape halved embryos. Cell length along apical-basal axis was measured at 8 hpf control blastula (which is equivalent to flat shape halves), 10 hpf control blastula (equivalent to cup shape halves), control 14 hpf blastula (equivalent to blastula halves) and cup shape halves and compared between them. Cell length of cup shape halves was significantly longer than the cell length of control blastula at any timing. (Tukey HSD, $p < 0.01$). Source data are provided as a Source Data file.

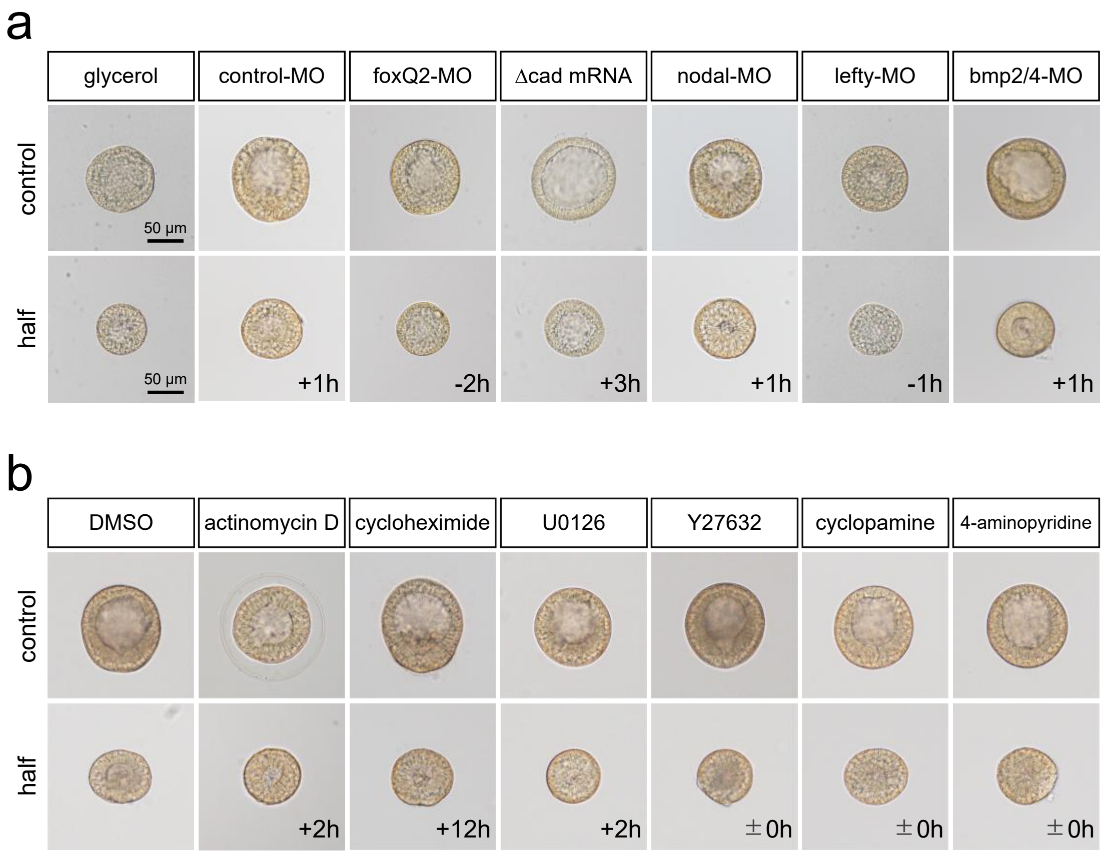

Figure S4 | Signaling pathways which is essential for body axes formation are not involved in the sphere formation. (a) Signaling molecules that is critical for sea urchin anterior-posterior and dorsal-ventral axis formation were inhibited with morpholino antisense oligo (MO) against foxQ2, nodal, lefty, and bmp2/4. Canonical wnt pathway were inhibited by overexpression of Δcadherin mRNA. (b) Transcription, translation, MAPK signaling pathway, Rho signaling pathway, hedgehog signaling pathway, and K^+ ion channel was inhibited with actinomycin D, cycloheximide, U0126, Y27632, cyclopamine and 4-aminopyridine, respectively. The time written in the corner of each picture indicates the average time difference for sphere formation compared to the control (glycerol or DMSO). Detail protocol for each reagent treatment is written in materials and methods. Source data are provided as a Source Data file.

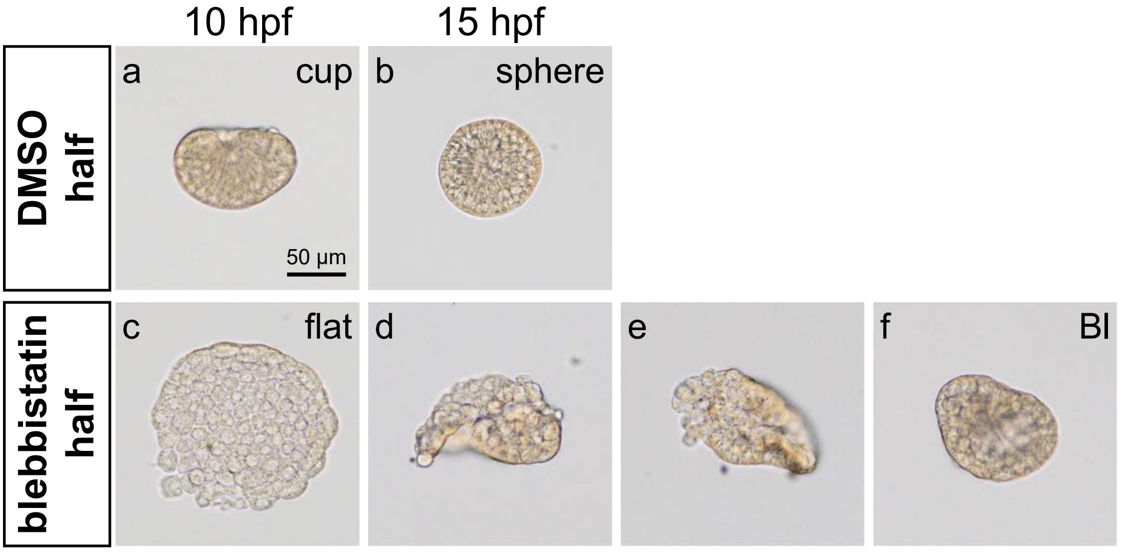

Figure S5 | Myosin-II activity inhibited halves become distorted sphere. (a, b) Halved control embryos. (c-f) Halved myosin-II activity inhibited embryos with blebbistatin-(-). Although blebbistatin treated halves finally formed blastula, sphere was irregularly distorted. 5 hours average delay was observed from the timing at which the control halves formed spheres. Developmental stage of each image is written in right corner. Hpf, hours post fertilization.

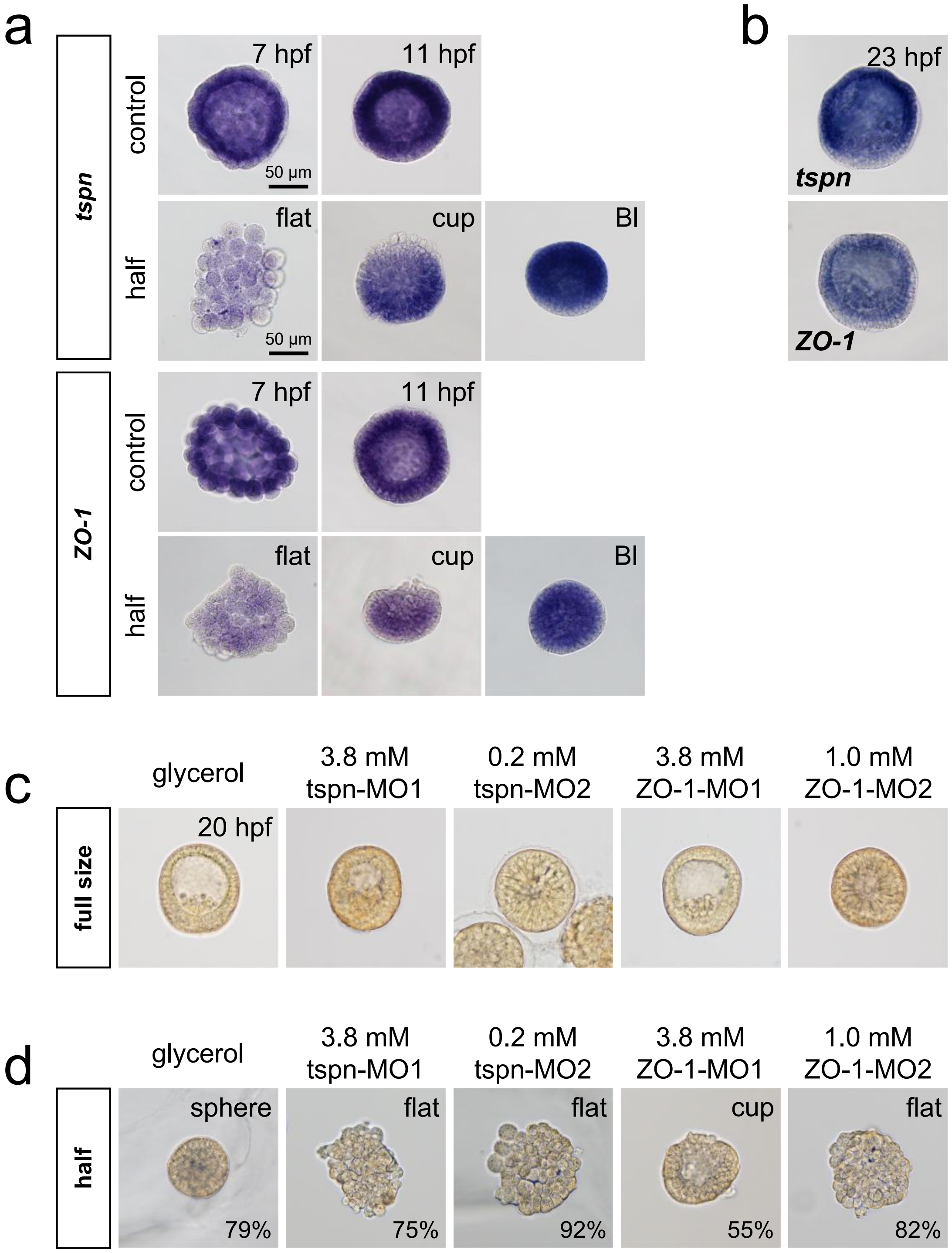

Figure S6 | Gene expression pattern and knockdown phenotype of septate junction associated gene. (a) *in situ* hybridization of tetraspanin (tspn) and ZO-1 in intact and halved embryos. (b) *in situ* hybridization of tspn and ZO-1 at mesenchyme blastula stage

in intact embryos. The signals were detected in the ectoderm except vegetal plate region in both tspn and ZO-1 probes, these signal patterns were consistent with the previous report in *S. purpuratus* (Jonusaite et al. 2023). (c) tspn and ZO-1 morphants phenotype in full-size embryos. Observation was carried out at the timing control embryos reached mesenchyme blastula stage. (d) Phenotype of tspn and ZO-1 morphants halves. Percentages indicate the ratio of individuals that showed the same phenotype with each photograph at the timing of observation. Developmental stage is written in right upper corner. Bl, blastula; Hpf, hours post fertilization. Source data are provided as a Source Data file.

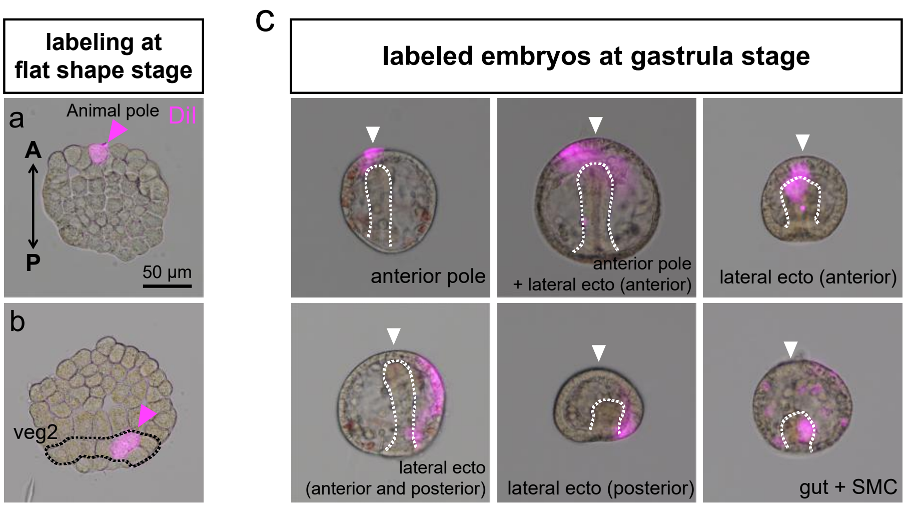

d

Labeled cell	Labeled cell position at gastrula stage						
	Anterior pole		Lateral ectoderm			Posterior pole	
	Anterior pole	Anterior pole + lateral ectoderm	Anterior half ectoderm	Both anterior and posterior ectoderm	Posterior half ectoderm	Gut + lateral ectoderm	Gut, SMC
Anterior pole (n=33)	21.2%	33.3%	15.2%	12.1%	18.2%	0%	0%
Veg2 linage (n=32)	0%	0%	0%	6.25%	12.5%	6.25%	75.0%

= Original fate of labeled cell

Figure S7 | Lineage trace of animal pole and veg2 cell in halved embryos. (a, b) Animal pole cell or veg2 linage cell were labeled with DiI at flat shape stage. Magenta arrowheads indicate the labeled cell. A, anterior; P, posterior. (c) Labeled cell position was checked at gastrula stage. Observed position are indicated at right corner in each image. White arrowheads, anterior pole; white dashed lines, outline of archenteron; ecto, ectoderm. (d) Result of lineage trace experiments. Grey shaded boxes indicate original fate of the labeled cell. SMC, secondary mesenchyme cell. Source data are provided as a Source Data file.

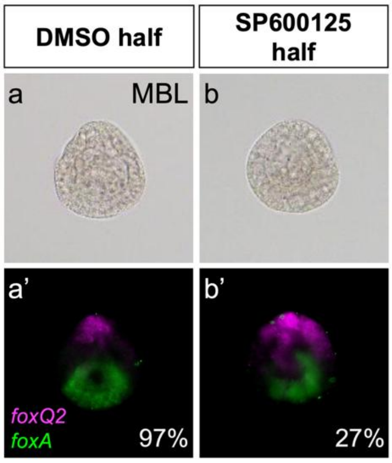

Figure S8 | Non canonical Wnt signaling is partially involved in anterior-posterior axis reformation in halved embryos. c-Jun N-terminal kinase (JNK) was inhibited with SP600125 in halved embryos from cup shape embryos (a,b), then gene expression of *foxQ2* and *foxA* were observed with *in situ* hybridization chain reaction (a',b'). Each percentage indicates that the ratio of embryos showed the same gene expression pattern with picture. 27% of SP600125 treated halves failed to re-form A-P axis. Source data are provided as a Source Data file.

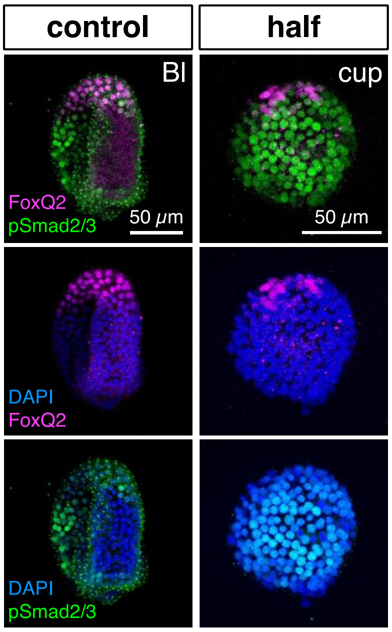

Figure S9 | Ectopically expressed Nodal functions in halved embryos. Immunofluorescent with phospho-Smad2/3 and FoxQ2 antibodies in intact and halved embryos. pSmad2/3 signals were ubiquitously observed except animal and vegetal pole in cup shape halved embryos, whereas pSmad2/3 signals were restricted to oral ectoderm in control. FoxQ2 signals were detected at the edge of cup in most of halved embryos. Developmental stage is written in right corner of the images. Bl, blastula.

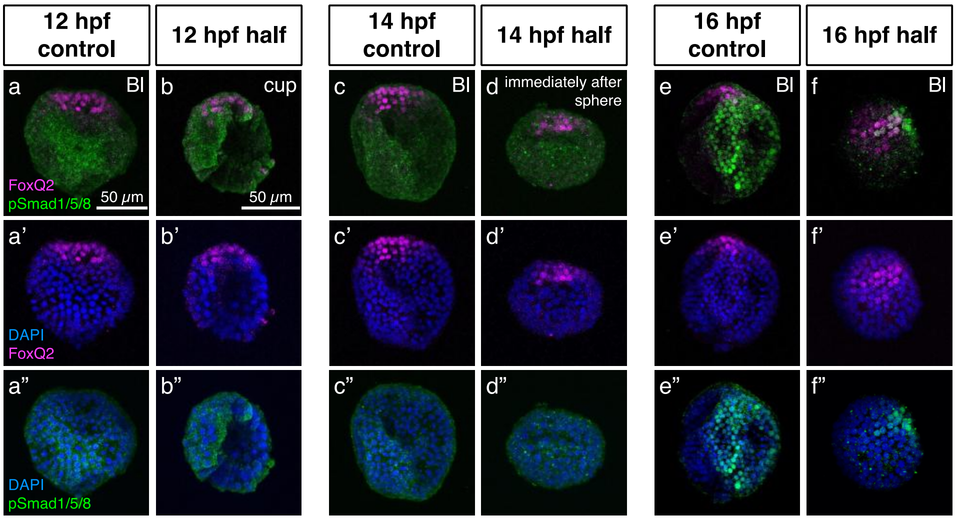

g

N=4	pSmad1/5/8 +	pSmad1/5/8 -
Cup	1%	99%
Almost sphere	2.5%	97.5%
Sphere	36%	64%
Blastula	82%	18%

Figure S10 | BMP2/4 starts function after sphere formed in halved embryos. (a-f) Immunofluorescent with phospho-Smad1/5/8 (pSmad1/5/8) and FoxQ2 antibodies in halved embryos at cup (b), immediately after becoming sphere formed (d) blastula (f) and corresponding timing of intact embryos (a, c, e). Developmental stage is written in right corner of the images. Bl, blastula. (g) Ratio of individuals which were observed pSmad1/5/8 signals at each stage in halved embryos. Source data are provided as a Source Data file.

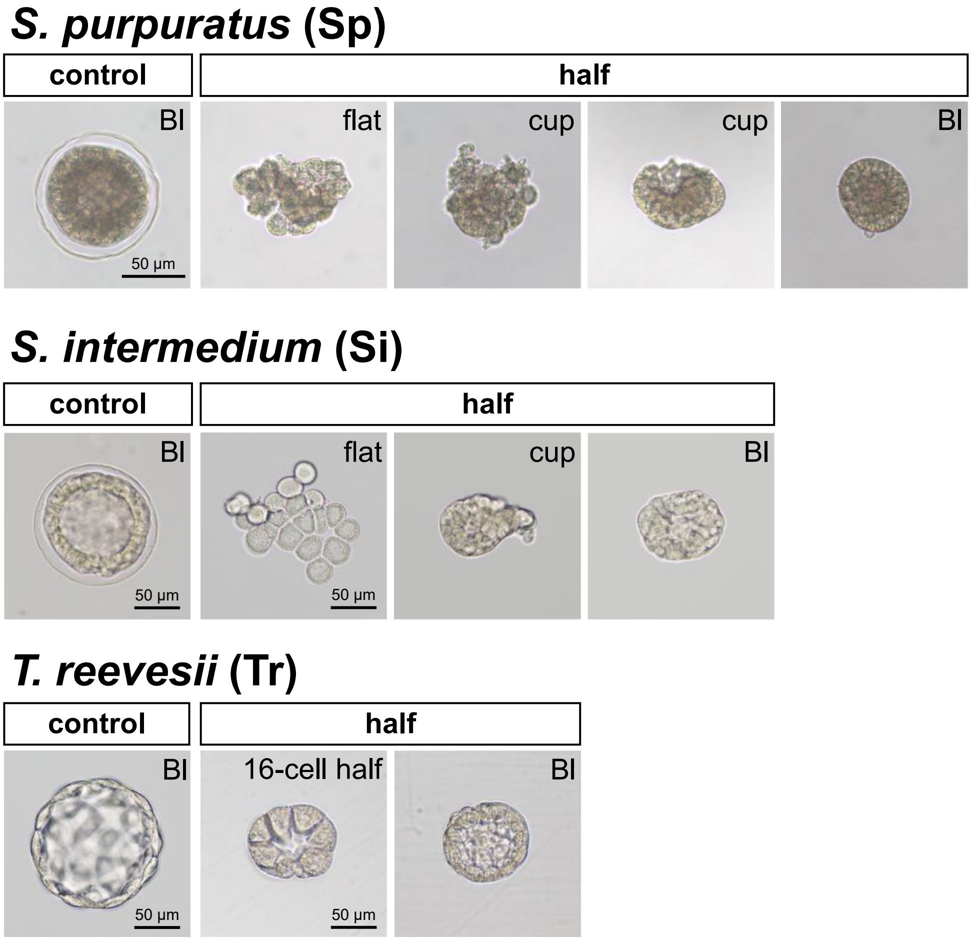

Figure S11 | Halved embryos development in various sea urchin species. Developmental process of 2-cell stage blastomeres of *Strongylocentrotus purpuratus* (Sp), *Strongylocentrotus intermedium* (Si) and *Temnopleurus reevesii* (Tr) were observed. Halved Sp and Si were developed in the same manner with *H. pulcherrimus*. On the other hand, halved Tr directly became blastula without through flat-cup stage. Developmental stage is indicated in the right corner. Bl, blastula.

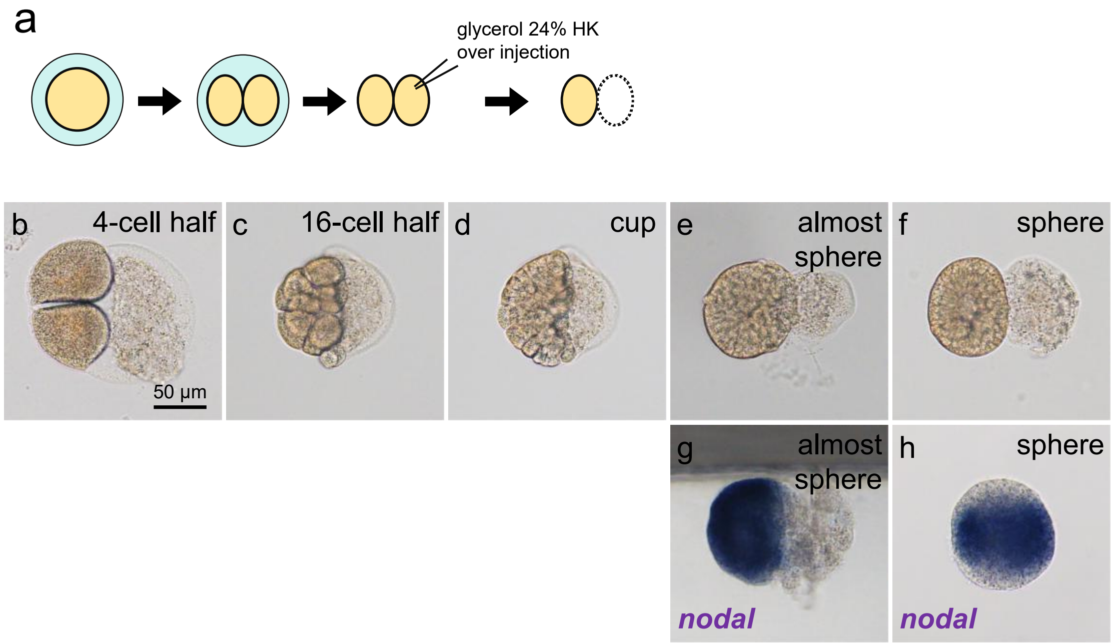

Figure S12 | Halved embryos make sphere even though the adhesion to adjacent half. (a) Schematic image of the experimental procedure. Fertilization envelope was removed at 2-cell stage, then one of the blastomere was killed by over injection of glycerol 24% HK buffer. Adhesion between the living blastomere and the killed blastomere is sustained. (b-f) Developmental process of the living halves which had adhesion to killed halves. Although the living halves behave as half body of full-size embryo until cup stage (b-d), eventually living halves form sphere shape independent from killed halves (e, f). (g, h) *in situ* hybridization with *nodal* probe. *nodal* expression patterns were identical to halved embryos that completely lost adhesion to the paired halves (see Figure 3p, q). Developmental stage of each image is written in right corner.

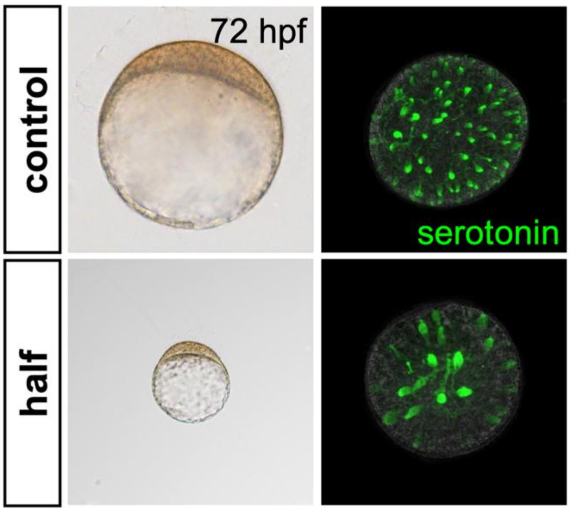

Figure S13 | Δcadherin mRNA–injected halves develop into permanent blastulae. At 72 hpf, both control and Δcadherin mRNA–injected halved embryos were examined. The halved embryos remained as permanent blastulae, and serotonergic neurons were widely observed, as in controls, likely due to the expansion of the anterior neuroectoderm caused by inhibition of Wnt/β-catenin signaling.
