# Supplementary material for: Unraveling the regulative development and molecular mechanisms of identical sea urchin twins
Source: Nat Commun. 2025 Sep 5;16:8005. doi: 10.1038/s41467-025-63111-z (PMC12413439; doi:10.1038/s41467-025-63111-z)
Supplement: Supplementary file 3 — Description of Additional Supplementary Files [file 41467_2025_63111_MOESM3_ESM.pdf]

Description of Additional Supplementary Files

File Name: Supplementary Movie 1
Description: This movie shows the transition of a halved embryo from flat to cup to sphere, as observed using light-sheet microscopy. Actin filaments are labeled with Lifeact-mCherry, and nuclei are stained with H2B-Venus.

File Name: Supplementary Movie 2
Description: Reactivation of the canonical Wnt pathway upon sphere formation in halved embryos, visualized by nuclear β-catenin-Venus. Cell membrane labeled with membrane-tagRFP.
